# Supplementary material for: The Remarkable Properties of Oil-in-Water Zein Protein Microcapsules
Source: Molecules. 2026 Jan 1;31(1):153. doi: 10.3390/molecules31010153 (PMC12787046; doi:10.3390/molecules31010153)
Supplement: Supplementary file 1 [file molecules-31-00153-s001.zip › molecules-3943688-supplementary.pdf]

Supplementary Materials

SM1. Preparation of o/w ZP/SO microcapsules

A set of experiments was designed to evaluate the system response while tuning several synthetic parameters. The list of the programmed experiments is reported in the Table S1. Each system was monitored for a minimum of 5 days to a maximum of 1 month. A 1 mL solution of 5 mg/mL ZP in EtOH/H_2O 70/30 (v/v) and 10 μL SO [1:100 oil/solution (v/v)] was selected as the standard solution for preparing o/w emulsions by ultrasonic assisted emulsification (UAE). Test tubes were always kept immersed in an ice bath during ultrasound treatment, and sonication was always carried out in continuous mode. All experiments were carried out in triplicate.

Table S1. List of experiments to evaluate the effect of the applied acoustic power, sonication time, and protein concentration on o/w emulsions from ZP EtOH/H_2O 70/30 (v/v) and soybean oil solutions. Sample volume: 1 mL; soybean oil: 10 μL; sonication frequency: 20 kHz; sonication in continuous mode, test tube in ice bath.

Sample	ZP concentration (mg/mL)	Time (s)	Power (W)
1	5	45	220
2	5	45	165
3	5	45	110
4	5	25	220
5	5	25	165
6	7.5	25	220
7	10	25	220

Acoustic power

To assess the effect of the applied acoustic power on the emulsification process, UAE was carried out on a 5 mg/mL ZP solution at 110 W, 165 W and 220 W sonication power for 45 s. The progressive sedimentation of precipitate on the bottom of the test tube was assessed after 12 hours from the synthesis, and increased within the hours. No discernible excess of oil could be noticed.

As regards the size distribution and stability, the smallest particles' size was achieved at the highest applied acoustic power (220 W). Compared to the lower power settings, *i.e.* 165 and 110 W, the particles obtained at 220 W were smaller from the initial moments of the synthesis and through the time. This can be explained by the increased shear stress caused by the increased acoustic power. It should be considered that an increased acoustic power may partially unfold or denature ZP, facilitating the formation of a protein shell at the interface, and thus increasing the stability of the oil droplets.

All the systems investigated underwent a marked particle size growth (2 to 3 times their initial size), and ended in a meta-stable condition after approximately 1 week of storage at room temperature in EtOH/H_2O 70/30 (v/v) (Figure S1). It could be also noticed that the droplet number density markedly decreased over time, mostly because of coalescence and precipitation of ZP/SO microcapsules.

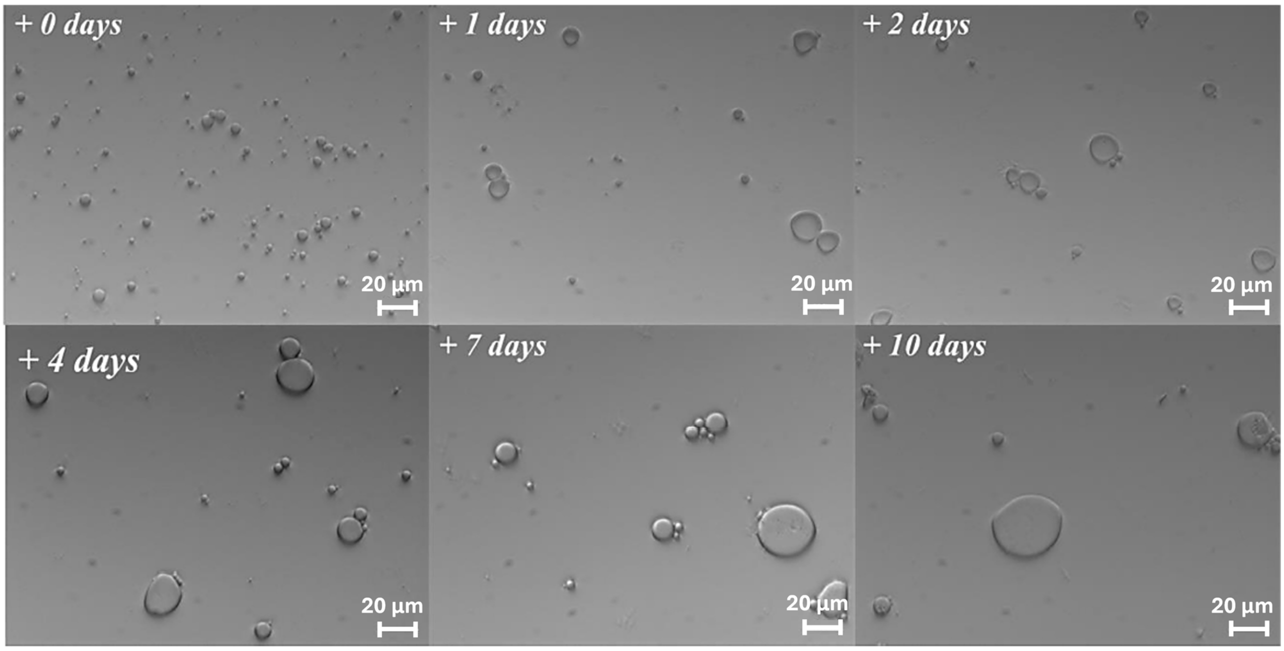

Figure S1. Optical microscopy images of o/w emulsions time progression from the day of the synthesis until +10 days. Sample: 5.0 mg/mL Zein EtOH/H_2O 70/30 (v/v) solutions and 10 µL soybean oil, sample volume = 1 mL. Sonication conditions: power = 220 W, time = 45 s, frequency = 20 kHz, continuous mode, test tube in ice bath.

Ultrasonication time

To assess the effect of the sonication time on the emulsification process, UAE on a 5 mg/mL ZP solution at 220 W and 165 W sonication power was carried out for 25 s and 45 s. It was observed that, under the lower applied power, reducing the duration of the sonication time led to a less homogeneous particle size distribution, and a marked drop of the particle number density. On the contrary, at the higher applied power (220 W) and 25 s sonication time, the particle size distribution appears to be more homogeneous. In addition, a shorter sonication time allowed the sample to reach a lower temperature during the ultrasound treatment, which could be a relevant criterion in the perspective of using this system to load an active ingredient. In conclusion, 25 s sonication time and 220 W applied power were selected as the best operative conditions (Figure S2). Nevertheless, 45 s sonication time constitutes a viable alternative option (see Figure S1 for comparison).

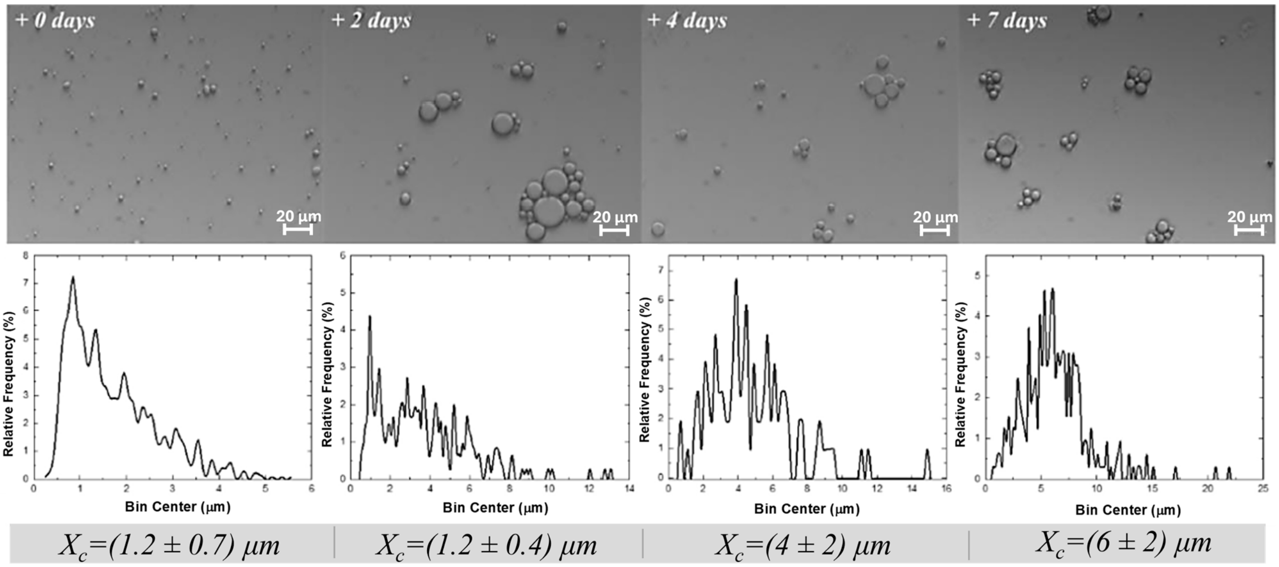

Figure S2. Optical microscopy images (scale bar: 20 µm) and size distributions of o/w ZP/SO microcapsules from the day of the synthesis until +7 days. Sample: 5.0 mg/mL ZP EtOH/H_2O 70/30 (v/v) solutions and 10 µL soybean oil, sample volume = 1 mL. Sonication conditions: power = 220 W, time = 25 s, frequency = 20 kHz, continuous mode, test tube in ice bath.

Protein concentration

The dependence of the UAE process on the protein concentration was investigated applying a power of 220 W for 25 s at a ZP concentration of 7.5 and 10 mg/mL. The increase in protein concentration led to a more homogeneous particle size, slower growth of the particle diameter, and a higher particle number density over time (Figures S3, S4). Best overall results were achieved at the higher protein concentration (10 mg/mL), which was also noticeably more stable over time. Quantitative measurements were carried out for 11 days from the synthesis. Optical microscopy images were collected until day 18 from the synthesis.

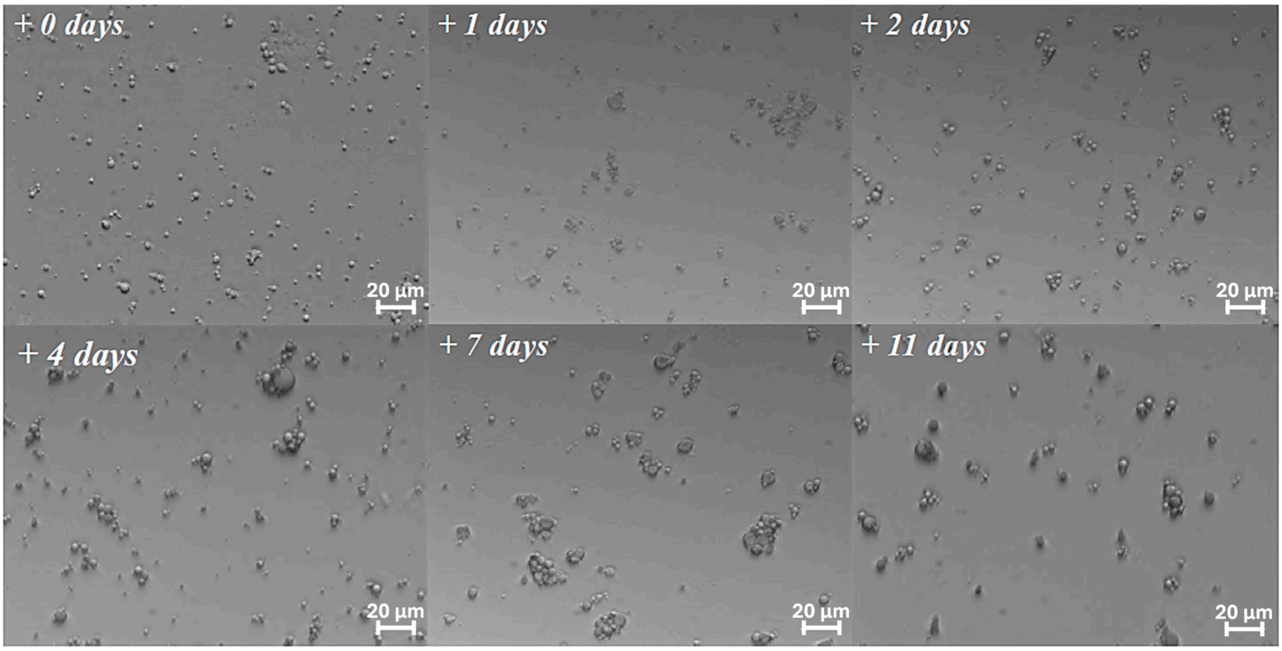

Figure S3. Optical microscopy images of o/w ZP/SO microcapsules from the day of the synthesis until +11 days. Sample: 10 mg/mL ZP EtOH/H_2O 70/30 (v/v) solutions and 10 mL soybean oil, sample volume = 1 mL. Sonication conditions: Power = 220 W, time = 25 s, frequency = 20 kHz, continuous mode, test tube in ice bath.

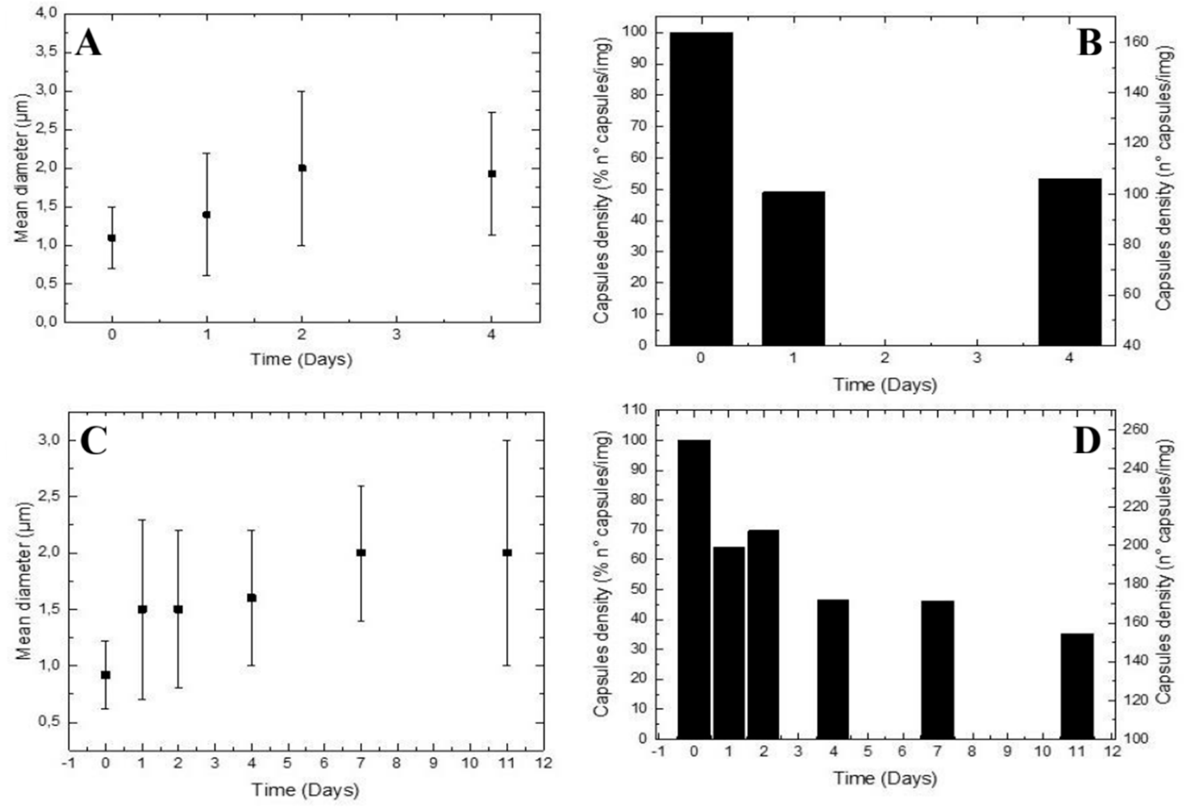

Figure S4. Time evolution of the mean diameter (A, C) and particle density (B, D) of ZP/SO microcapsules prepared from 7.5 mg/mL (A, B; 0-4 days) and 10.0 mg/mL (C, D; 0-11 days) of ZP in EtOH/H_2O 70/30 v/v. For all the samples: 10 µL soybean oil, sample volume = 1 mL; sonication: power = 220 W, time = 25 s, frequency = 20 kHz, continuous mode, test tube in ice bath.

ZP/SO ratio

Optical microscopy images of ZP/SO microcapsules obtained by UAE of ZP solutions of different concentrations (2.5 and 5 mg/mL) and different SO amounts (10, 25 and 50 µL) are reported in Figure S5. Sonication was carried out at frequency =20 kHz, power: 220 W, and in continuous mode for 25 s.

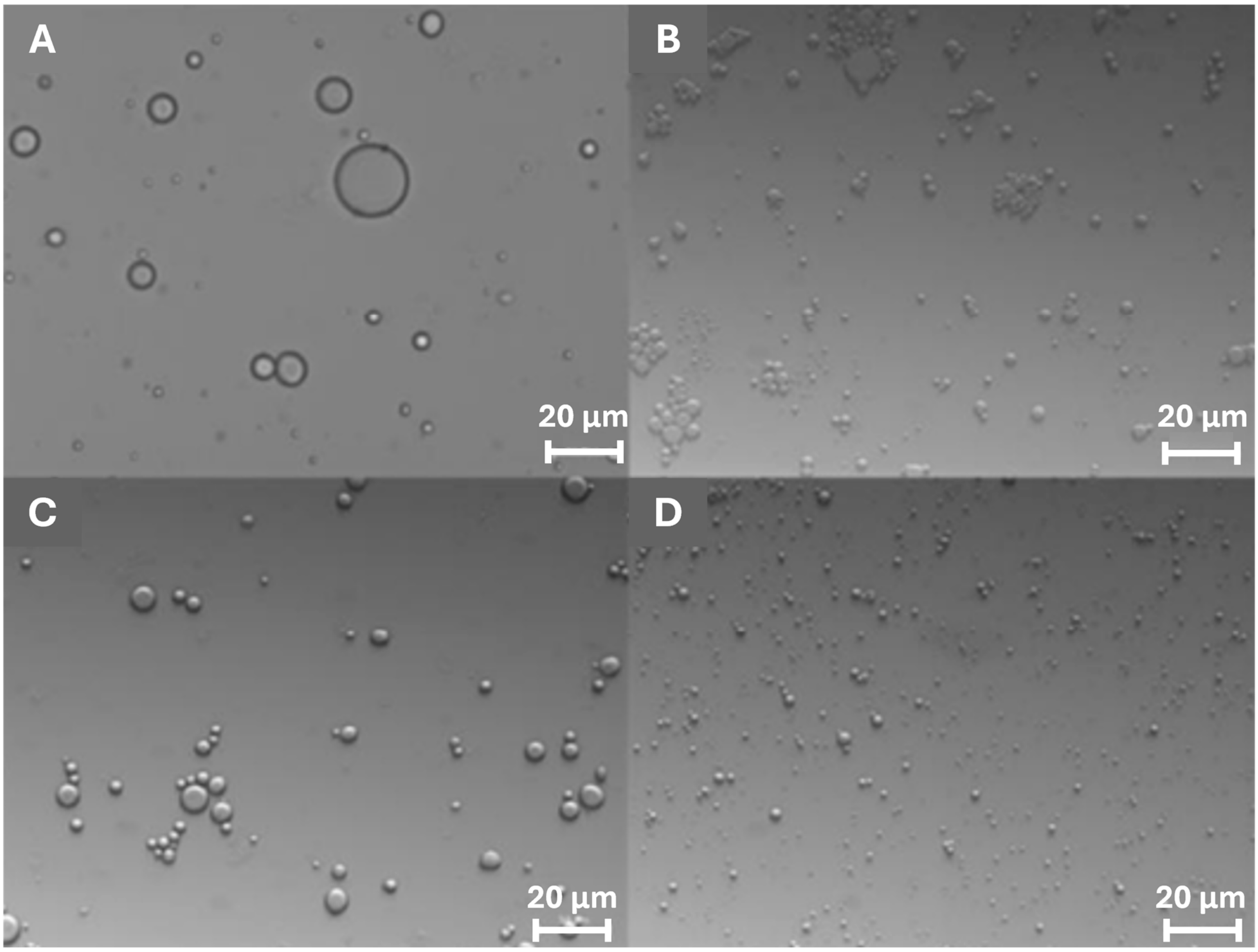

Figure S5. Optical microscopy images of o/w ZP/SO microcapsules. Sample volume: 1 mL; frequency: 20 kHz, power: 220W, continuous mode. A) 2.5 mg/mL ZP, 50 µL SO; B) 2.5 mg/mL ZP, 25 µL SO; C) 2.5 mg/mL ZP, 10 µL SO; D) 5 mg/mL ZP, 10 µL SO. Scale bar: 20 µm.

Storage

ZP Samples of 5.0, 7.5 and 10.0 mg/mL protein concentration were emulsified, with a sonication time of 25 s and an acoustic power of 220 W. Each system was split into two aliquots, one stored at room temperature and the other stored in a refrigerated environment at 4-5 °C. Higher coalescence at lower temperature was observed due to the reduced Brownian motion. As a result, contact and pressure between the particles are increased, which would promote interactions (aggregation) and, eventually, the rupture of the protective protein layer (increased coalescence of the particles). Dry storage under vacuum of the system favors its integrity. Interestingly, re-dispersion in solutions characterized by a higher H_2O content [i.e., EtOH/H_2O 10/90 (v/v) solution and 100% water] was easily carried out. The time dependence of the mean diameter and density of dried ZP/SO MC re-dispersed in EtOH/H_2O 70/30 (v/v) were reported in Figure S6 from 0 to 6 days.

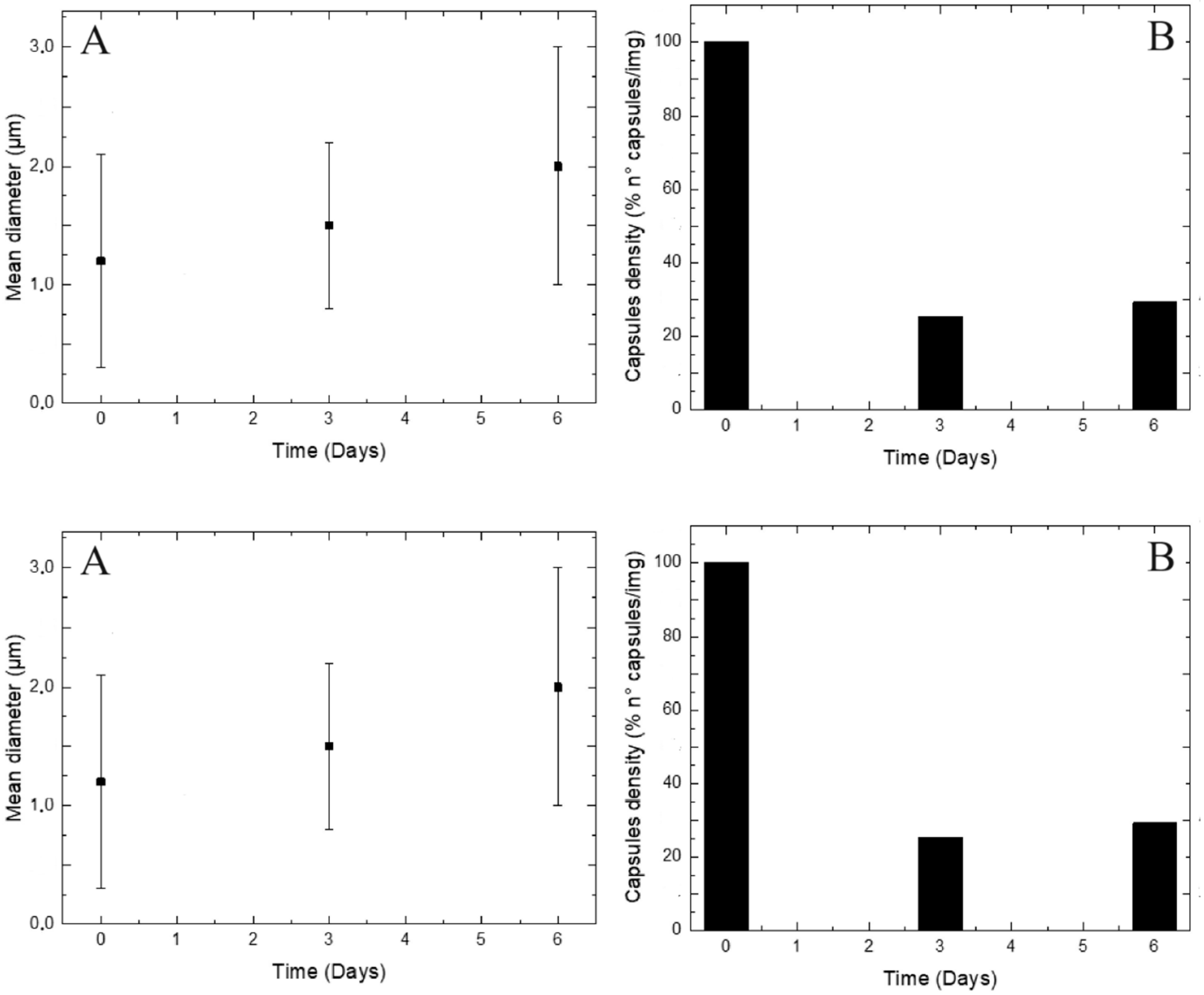

Figure S6. Time dependence of the mean diameter (A) and density (B) of o/w ZP/SO microcapsules, from the day of re-solubilization (0) to 6 days**.** Sample: 10 mg/mL ZP EtOH/H_2O 70/30 (v/v) solutions and 10 µL soybean oil, sample volume = 1 mL. Sonication conditions: power = 220 W, time = 25 s, frequency = 20 kHz, continuous mode, test tube in ice bath, then vacuum dried and solubilized in 2 mL of EtOH/H_2O 70/30 (v/v) solution.

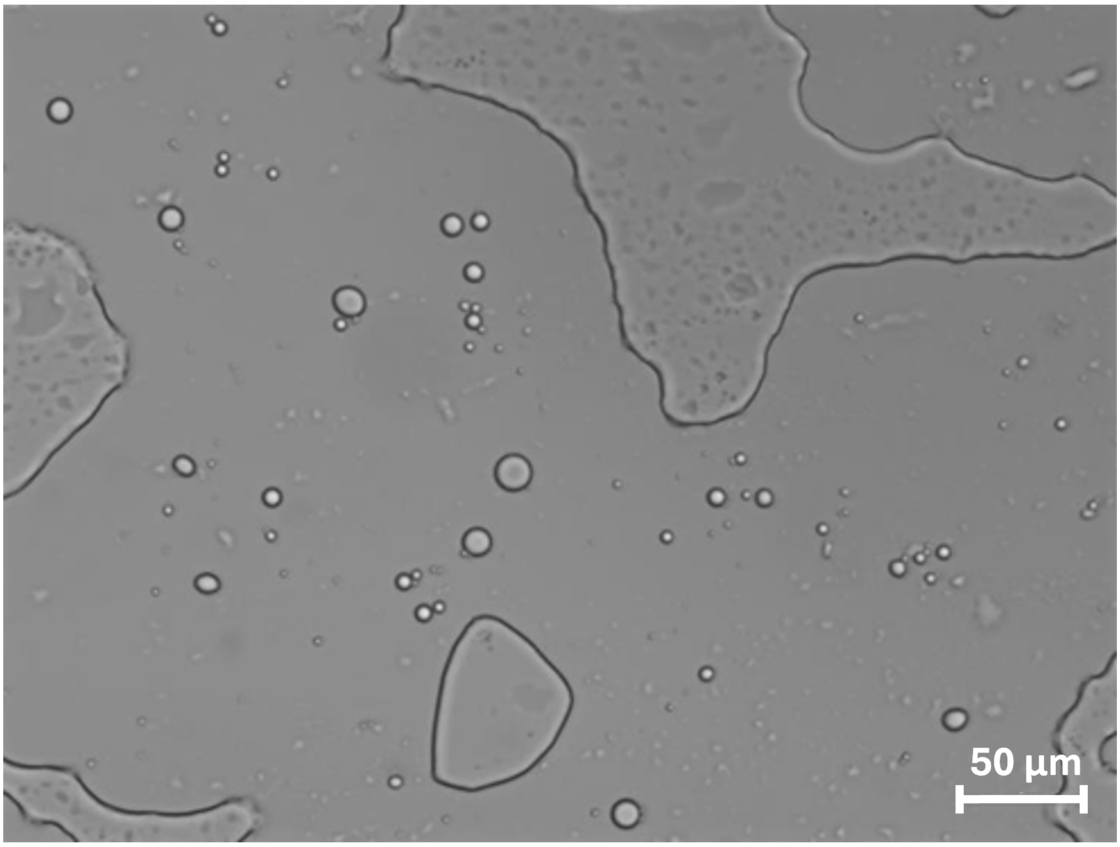

Figure S7. Optical microscopy images of o/w emulsions obtained by sonication of an EtOH/H_2O 70/30(v/v) and 50 µL soybean oil dispersion.

SM2. Morphological characterization of o/w ZP/SO microcapsules

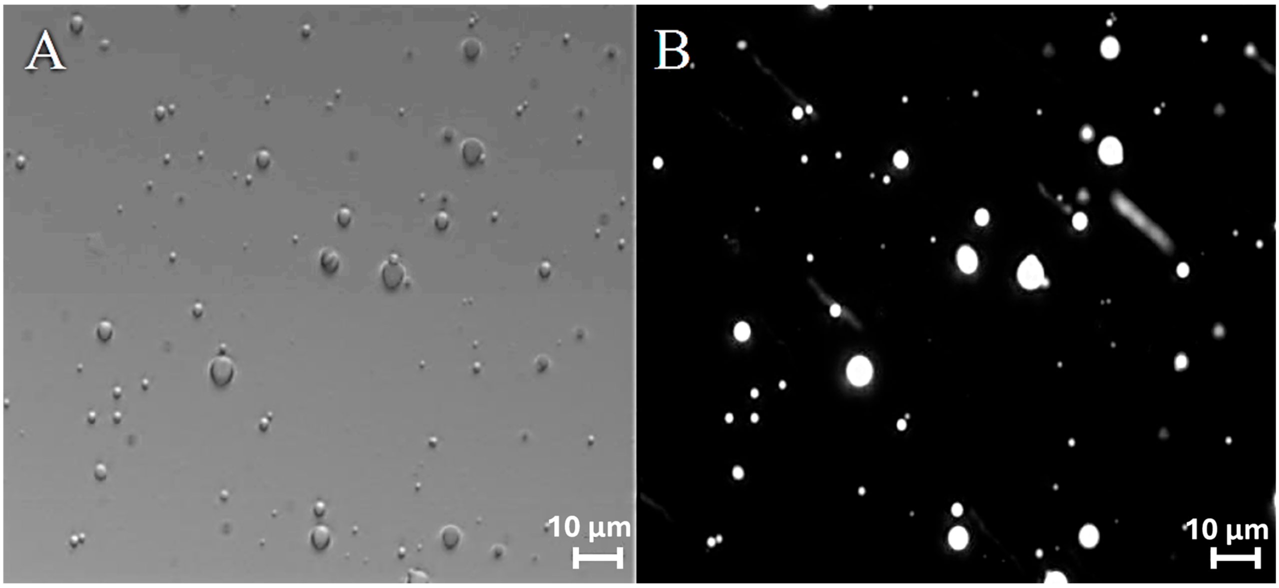

Figure S8. Bright-field (left) and fluorescence (right) microscopy images of o/w microcapsules prepared by UAE of 5.0 mg/mL ZP EtOH/H_2O 70/30 (v/v) solution and 10 µL soybean oil. Nile Red was included in soybean oil prior to the microcapsule preparation. Sample volume: 1 mL; sonication conditions: frequency = 20 kHz; power = 220 W; continuous mode.

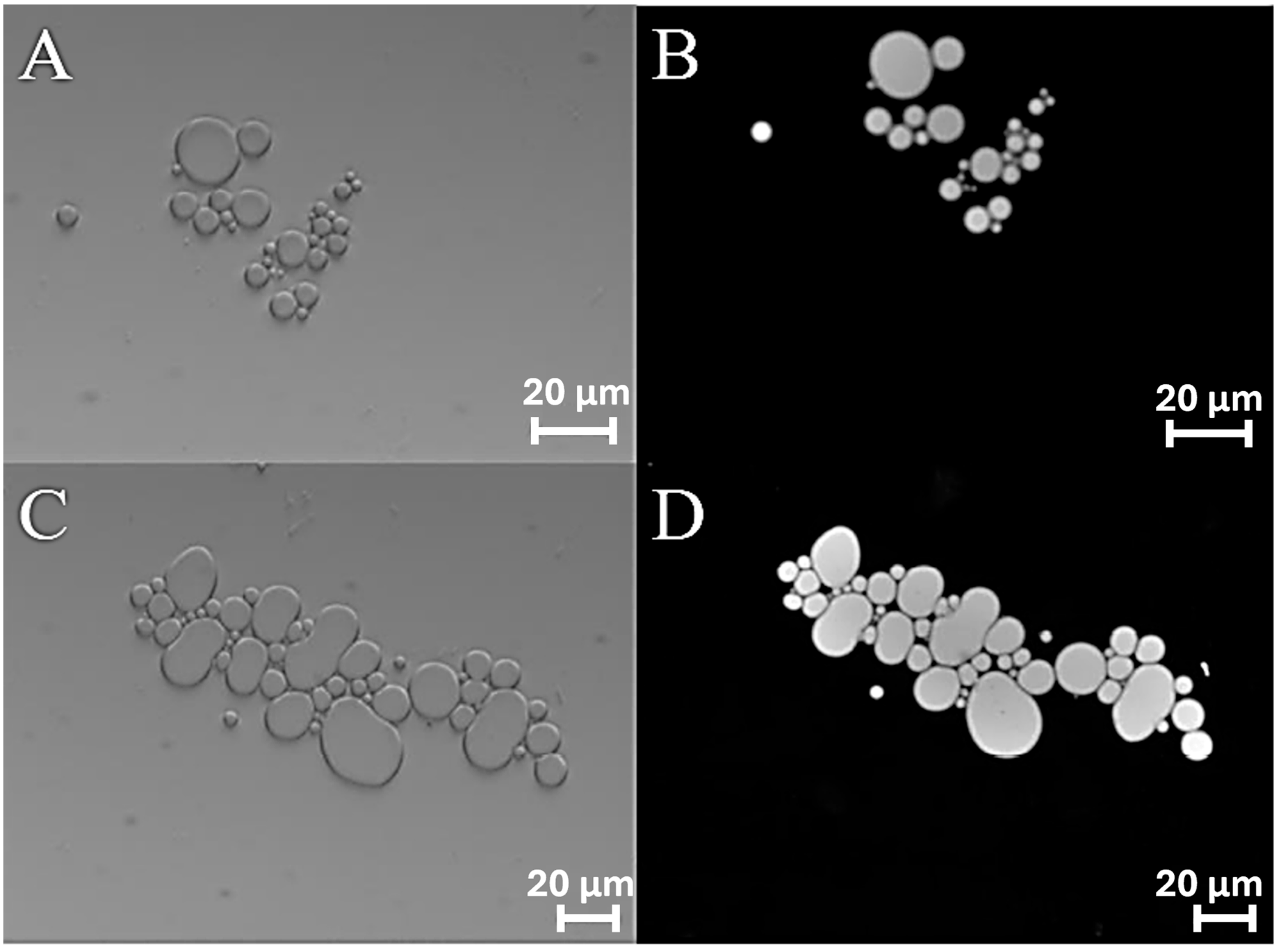

Figure S9. Bright-field (sx) and fluorescence (dx) microscopy images of o/w microcapsules (5.0 mg/mL ZP in EtOH/H_2O 70/30 (v/v) and 10 µL SO solution). Nile Red was included in SO prior to the microcapsule preparation. Particle clusters are imaged after +4 days from the synthesis. Sample volume: 1 mL; sonication conditions: frequency = 20 kHz; power: 220 W; continuous mode.

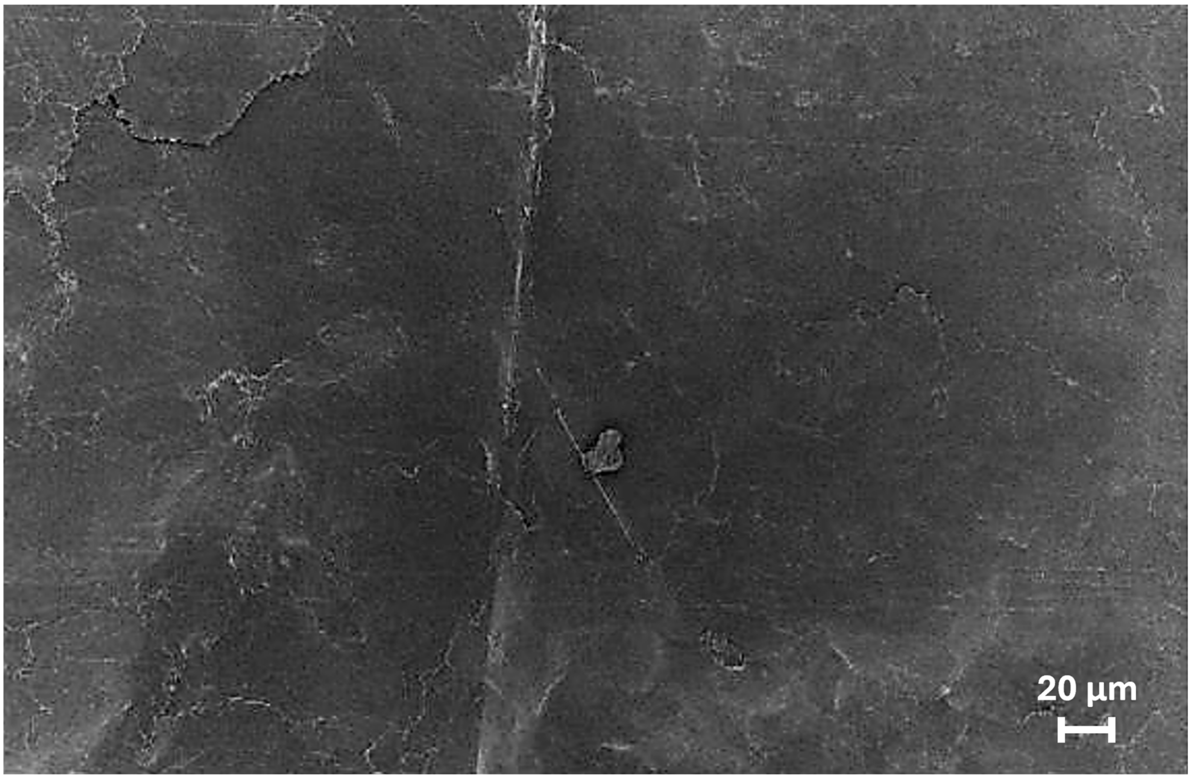

Figure S10. FE-ESEM image of o/w emulsions formed by EtOH/H_2O 70/30 (v/v) and 10 µL soybean oil. Sample volume: 1 mL; sonication conditions: Power = 220 W, time = 25 s, frequency = 20 kHz; continuous mode.

SM3. Optical spectroscopy characterization of ZP/SO microcapsules

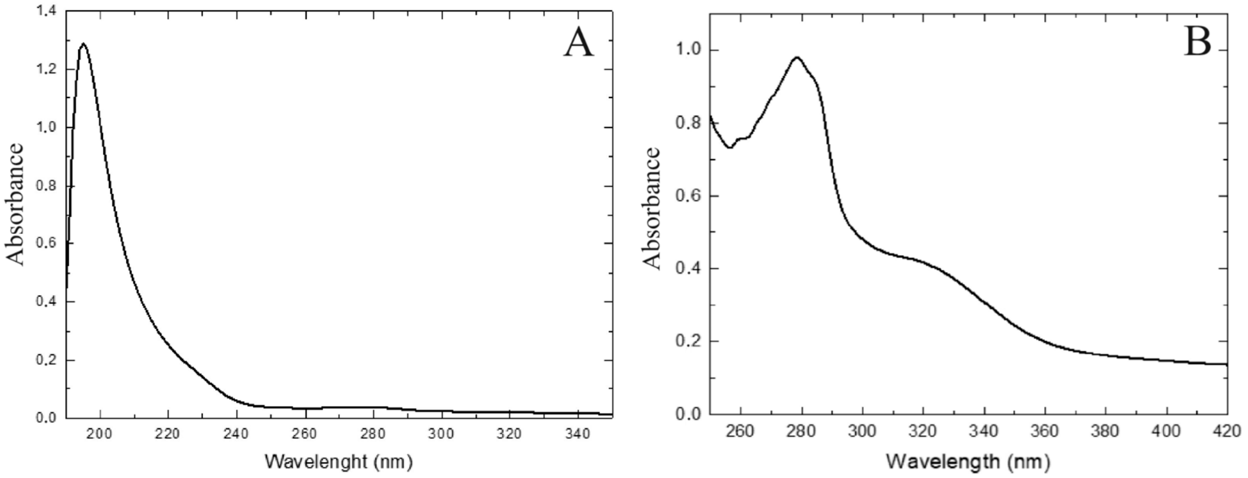

Figure S11. UV-Vis spectra of ZP in EtOH/H_2O 70/30 (v/v). A) 0.1 mg/mL (190-350 nm); B) 0.9 mg/mL (250-420 nm).

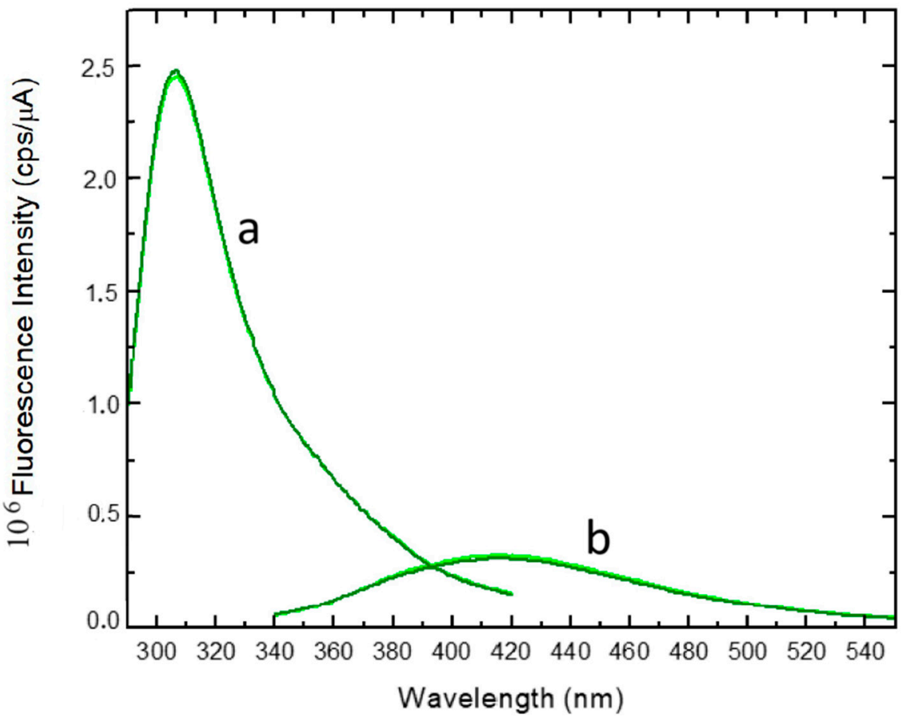

Figure S12. Fluorescence emission spectra of ZP in EtOH/H_2O 70/30 (v/v): a) λ_{ex}=283 nm; b) λ_{ex}=315 nm.

SM4. Stability of ZP/SO microcapsules under Temperature and pH stimuli

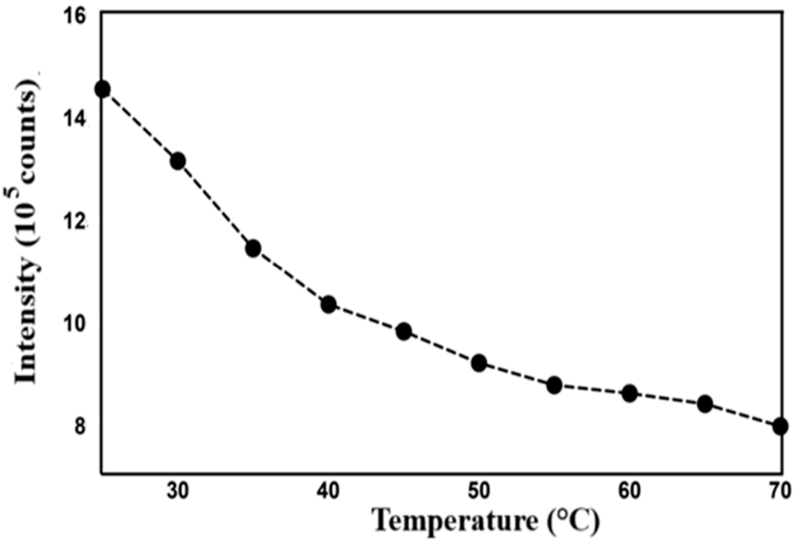

Figure S13. Rayleigh Light Scattering intensities of ZP/SO microcapsules at different temperatures.

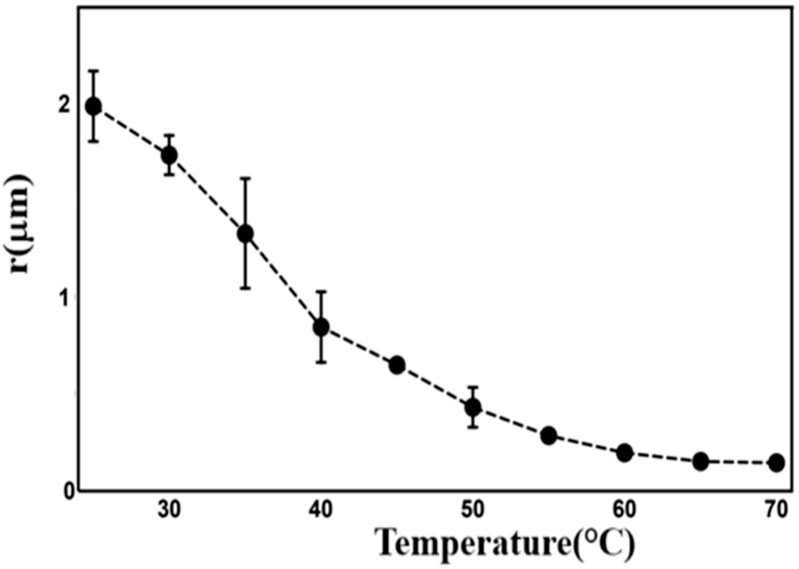

Figure S14. Hydrodynamic radius of ZP/SO microcapsules at different temperatures from Dynamic Light Scattering experiments.

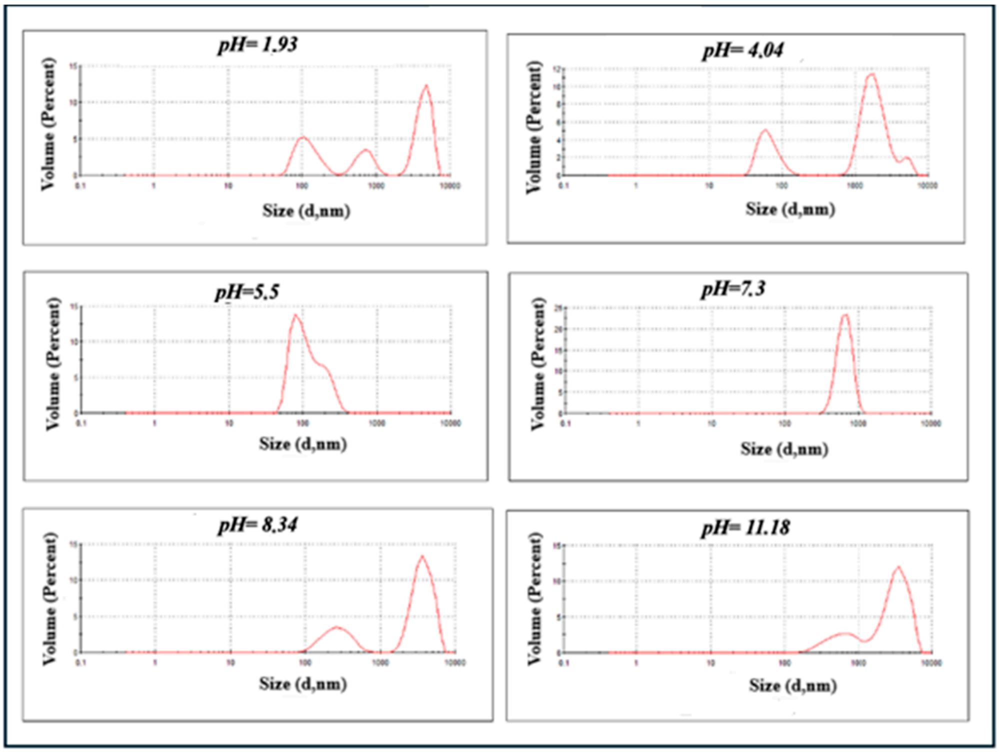

Figure S15. Size distribution (volume vs. diameter) of ZP/SO microcapsules at different pH's obtained by Dynamic Light Scattering measurements.

SM5. Spectroscopy characterization of Curcumin in solution and into ZP/SO microcapsules

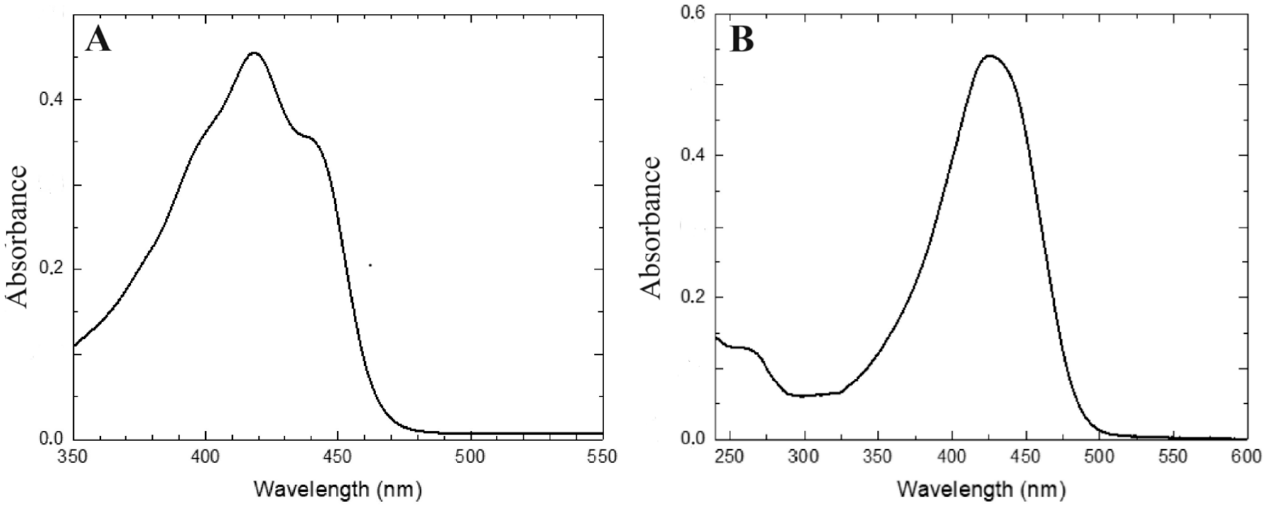

Figure S16. UV-Vis absorption spectra of Curcumin at 25°C: A) soybean oil; B) ethyl alcohol.

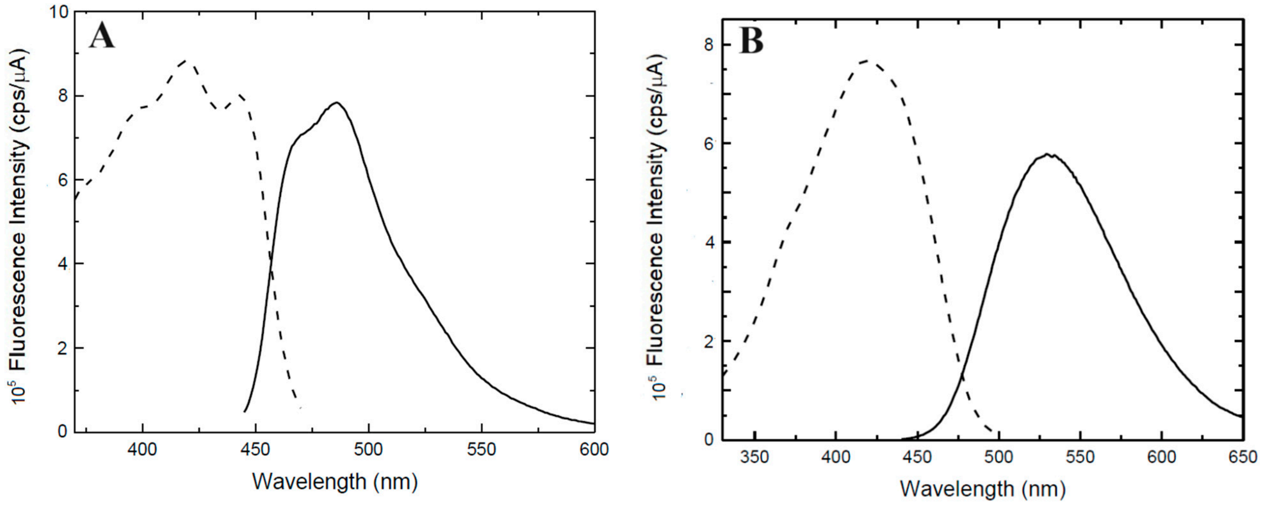

Figure S17. Fluorescence emission and excitation spectra of Curcumin at 25°C. A) Soybean oil; solid line: emission spectrum (λ_{ex}=440 nm), dashed line: excitation spectrum (λ_{em}= 487 nm). B) Ethanol; solid line: emission spectrum (λ_{ex}=425 nm), dashed line: excitation spectrum (λ_{em}= 530 nm).

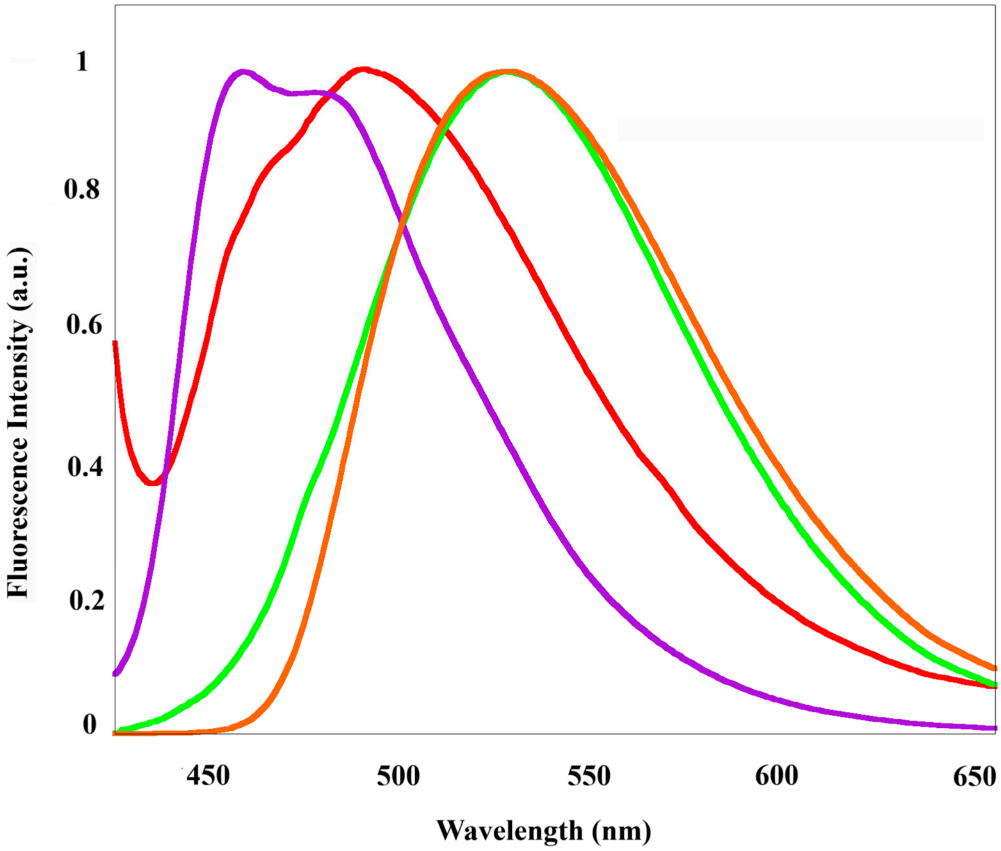

Figure S18. Fluorescence emission spectra at 25°C of Curcumin in soybean oil (purple line), in ZP/SO microcapsules (red line), in ethanol (green line) and EtOH/H_2O 70/30 (v/v) (brick red line).

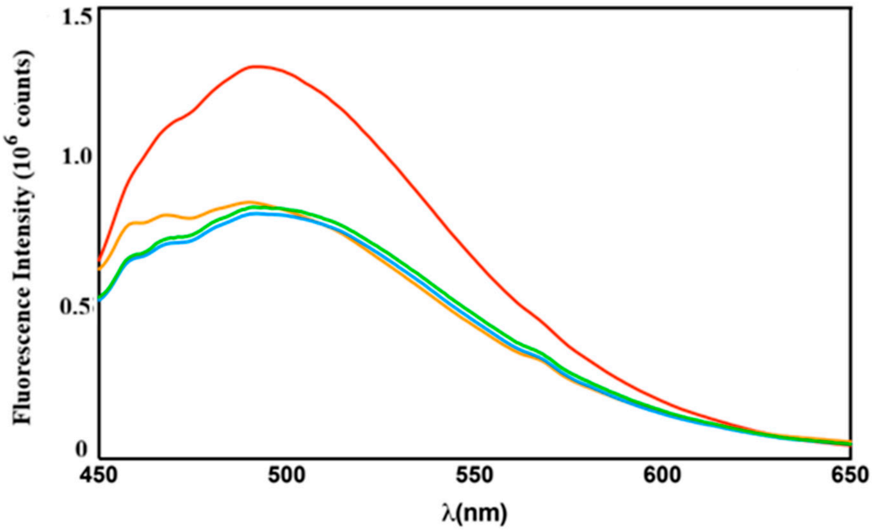

Figure S19. Fluorescence emission spectra of Curcumin in ZP/SO microcapsules at different pH's. Red line: pH=5.5; ocher: pH=4.3; green: pH=2.9; light blue: pH= 1.7.

SM6. Kinetic analysis of Curcumin release from ZP/SO microcapsules through Weibull model

Fluorescence emission intensity decays were reproduced by using the Weibull equation, adapted by Langenbucher [1,2] to dissolution/release process:

$$m = 1 - \exp\{1- [(t-t_i)^b/a]\} \qquad (S1)$$

where 'm' is the accumulated fraction of drug in solution, 'a' a scale parameter defining the time scale of the process, 't_i' the lag time before the onset of the release process, and 'b' the shape parameter. When b is less than 1, the shape of the curve is parabolic, as typical of a site-specific biphasic release kinetic [3].

The parameters obtained by fitting the experimental data at the investigated temperatures through equation (1) are reported in Table S2, while the comparison between experimental and computed decays at the investigated temperatures is shown in Figure S20.

Table S2. Fitting parameters obtained by reproducing the Curcumin fluorescence emission intensity decays at the different temperatures through the Weibull equation.

T(°C)	a	b	t_i(s)	χ^2
32	25.9	0.64	1.7	0.99
35	16.9	0.61	6.6	1
37	10.7	0.55	7.6	0.99
40	17.0	0.63	7.6	1

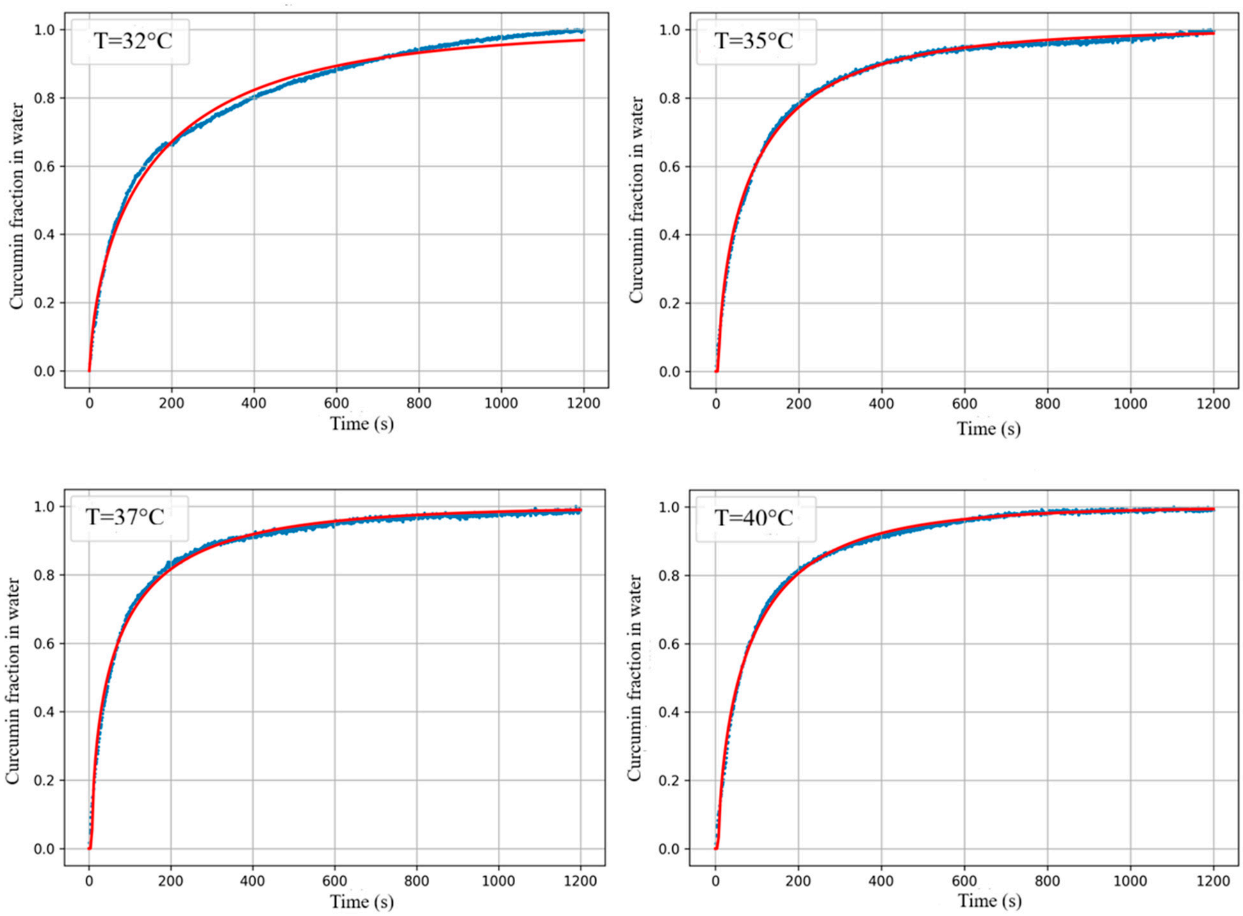

Figure S20. Fitting of the experimental Curcumin fractions in EtOH/H_2O solutions at different temperatures (blue curves) by the Weibull logistic model (red curves).
